# Supplementary material for: Structure Activity Relationship and Molecular Docking of Some Quinazolines Bearing Sulfamerazine Moiety as New 3CLpro, cPLA2, sPLA2 Inhibitors
Source: Molecules. 2023 Aug 14;28(16):6052. doi: 10.3390/molecules28166052 (PMC10460087; doi:10.3390/molecules28166052)
Supplement: Supplementary file 1 [file molecules-28-06052-s001.zip › supplemenrty figures S1-S15.pdf]

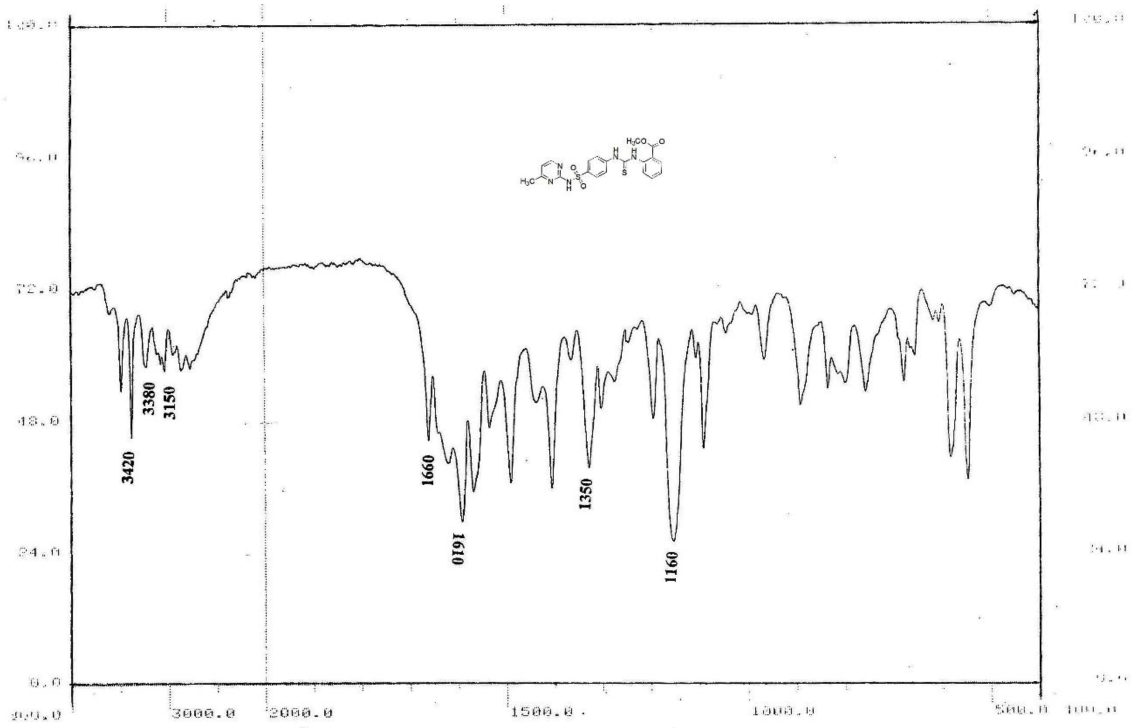

IR chart of compound 3

Figure S1: IR spectrum of comound **3**.

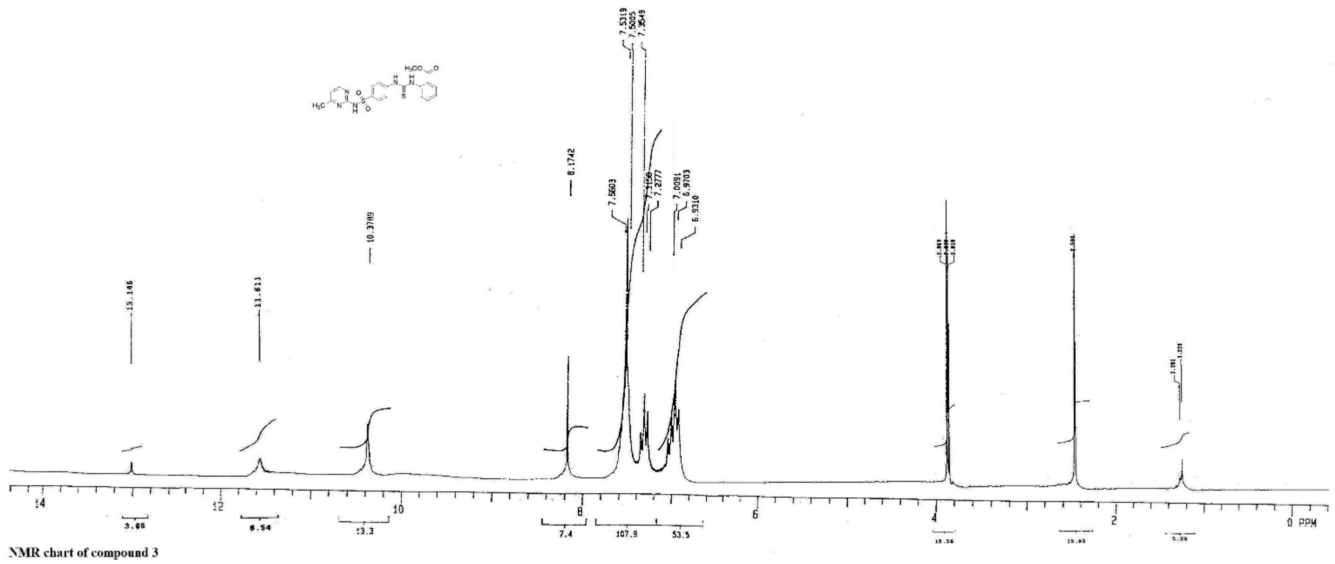

NMR chart of compound 3

Figure S2: ^{1}H-NMR spectrum of comound **3**.

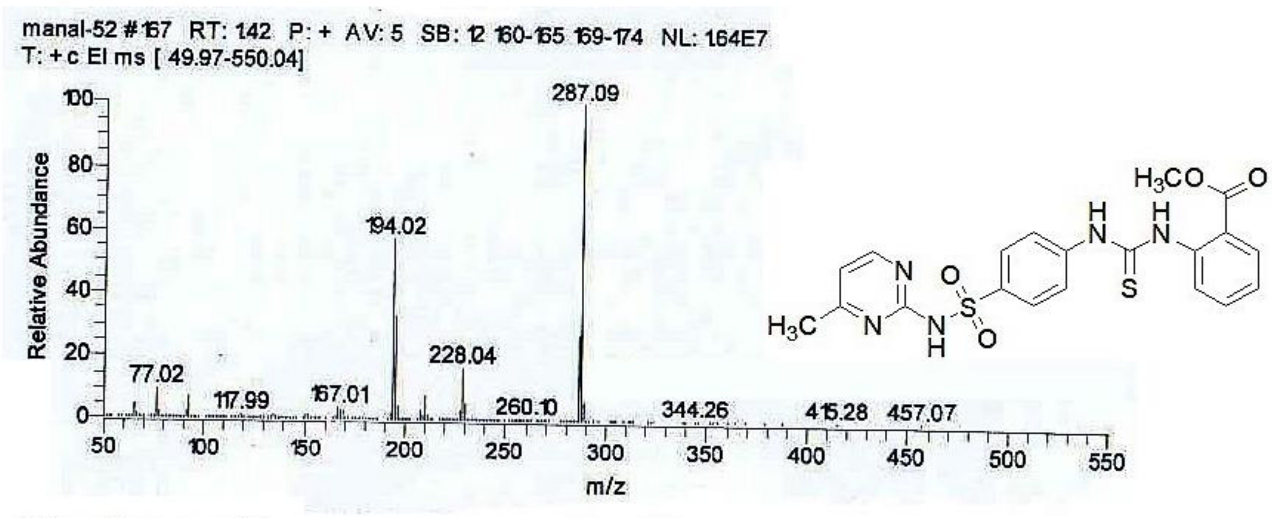

Mass of compound 3

Figure S3: Mass spectrum of comound **3**.

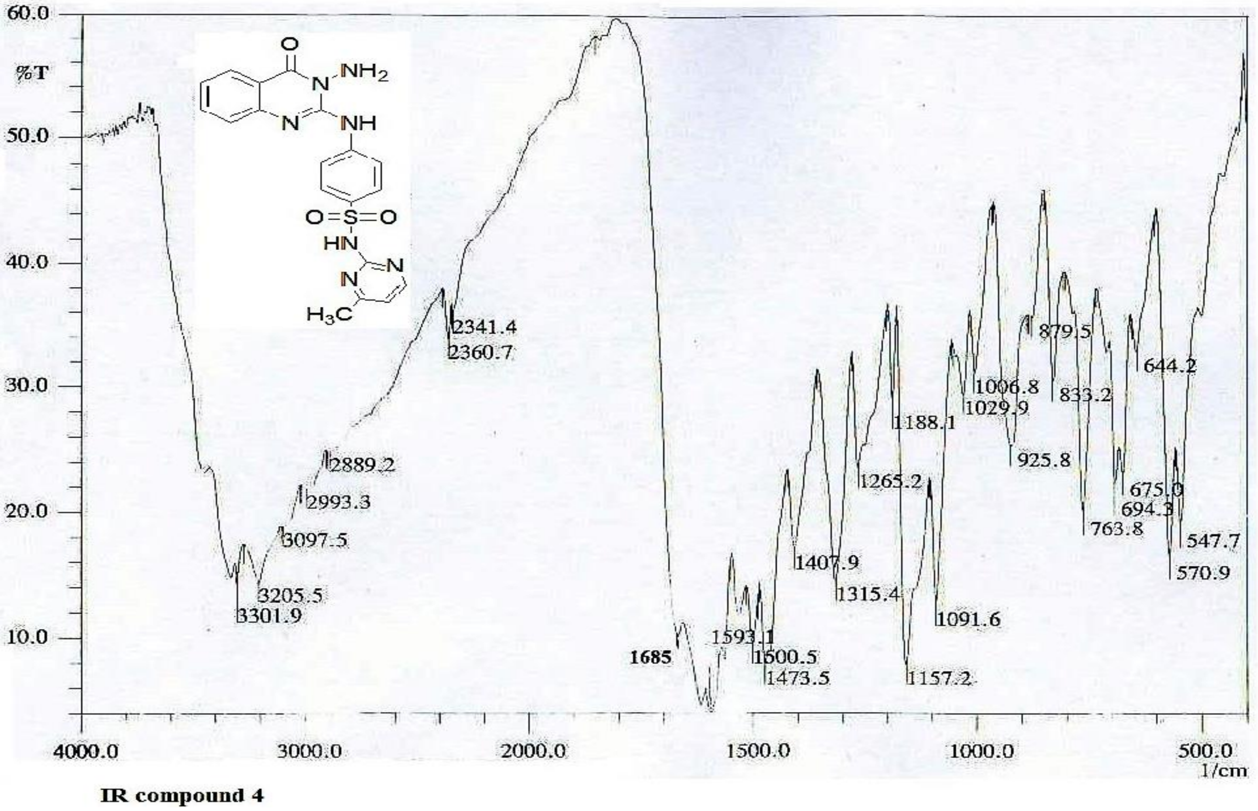

Figure S4: IR spectrum of comound **4**.

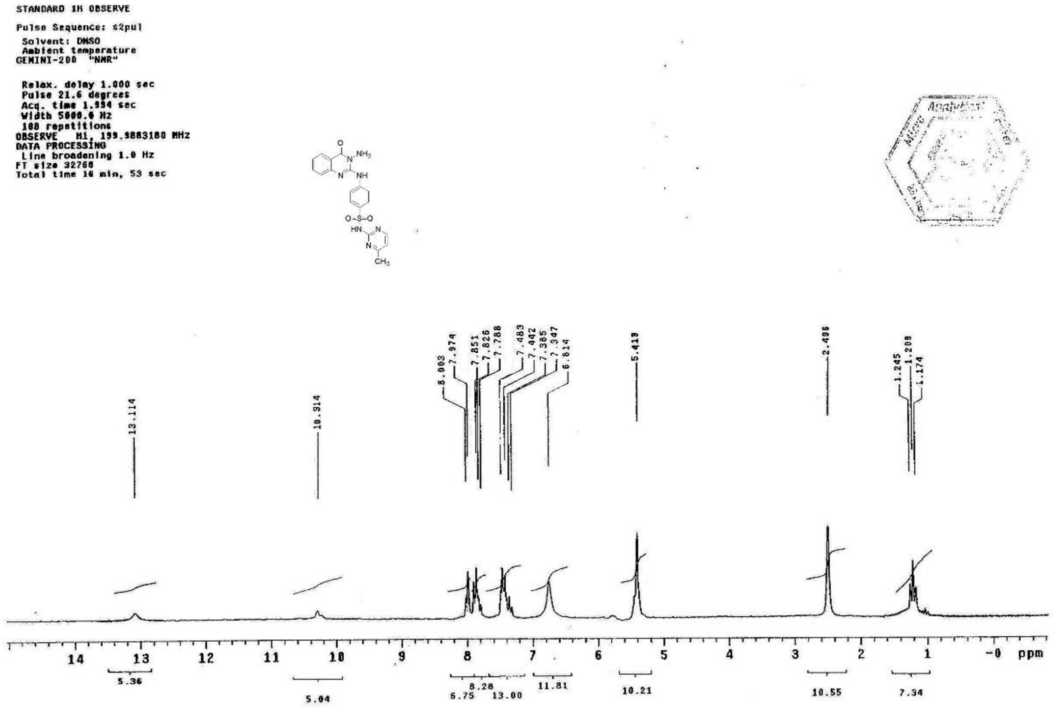

Figure S5: ^{1}H-NMR spectrum of comound **4**.

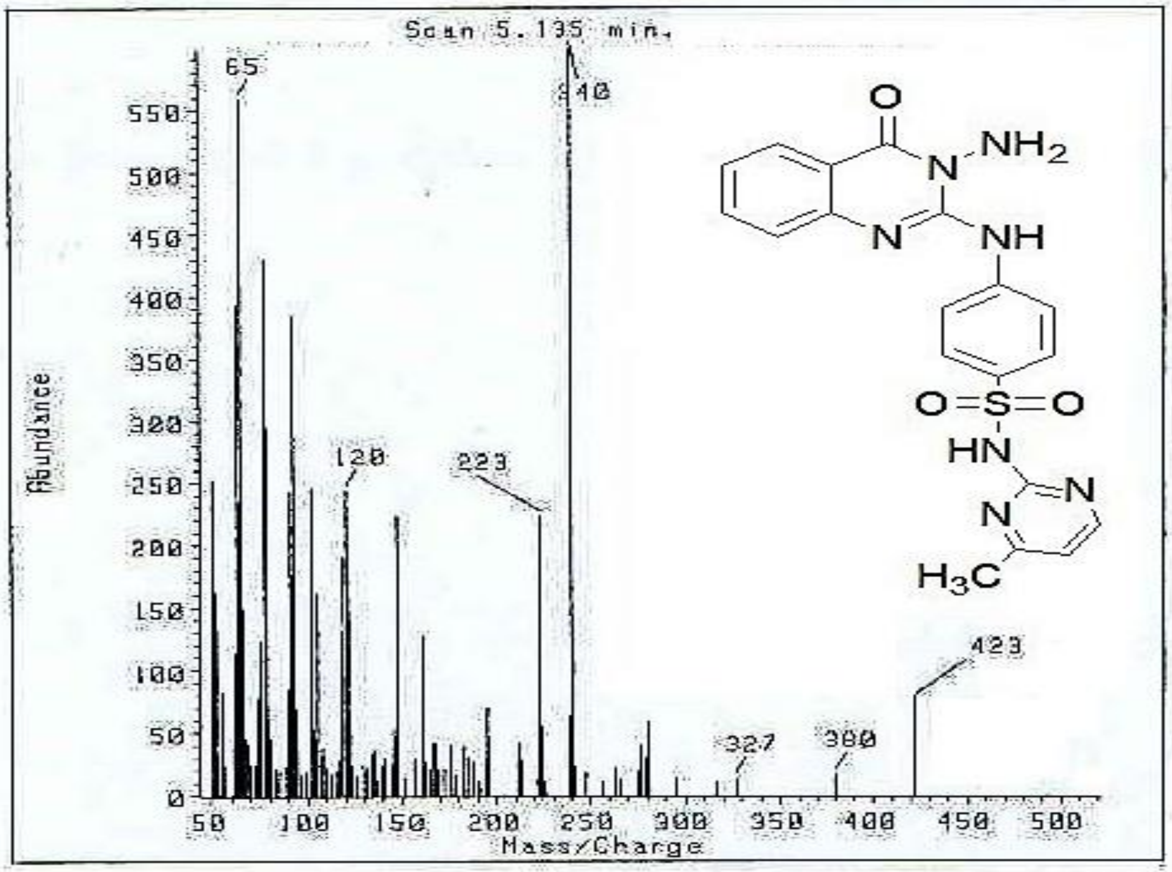

Mass of compound 4

Figure S6: Mass spectrum of comound **4**.

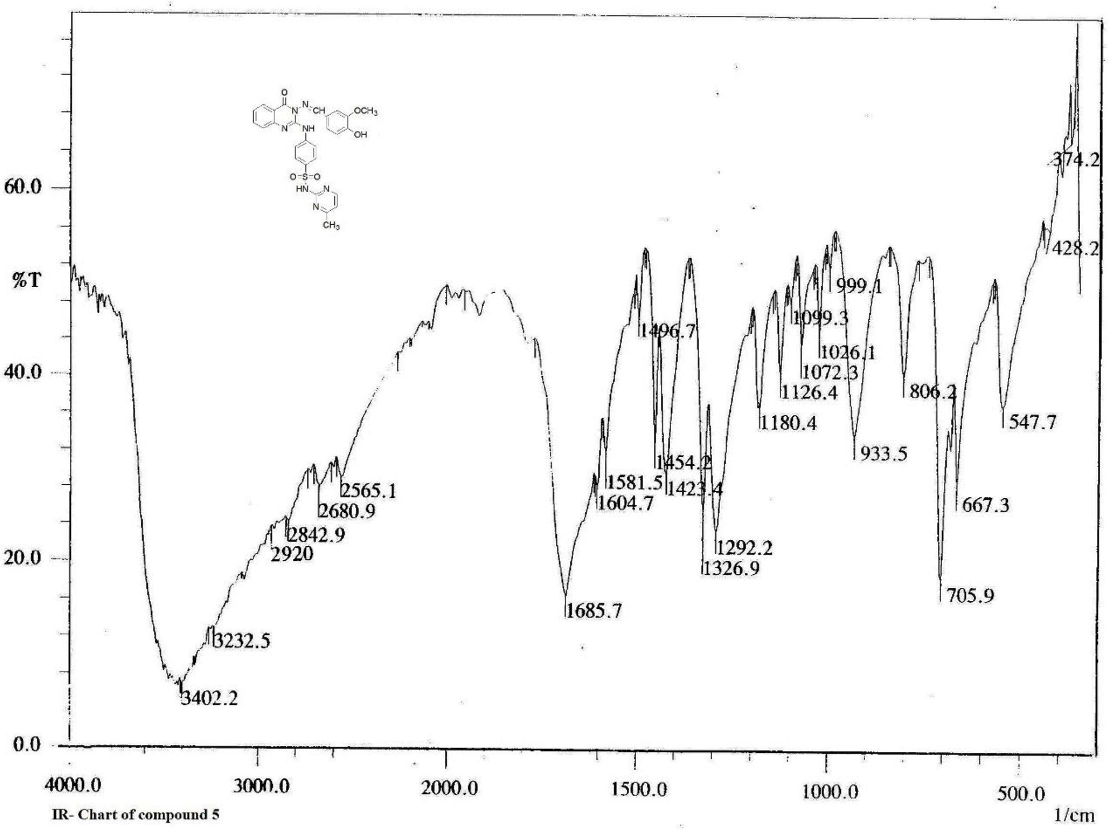

Figure S7: IR spectrum of comound **5**.

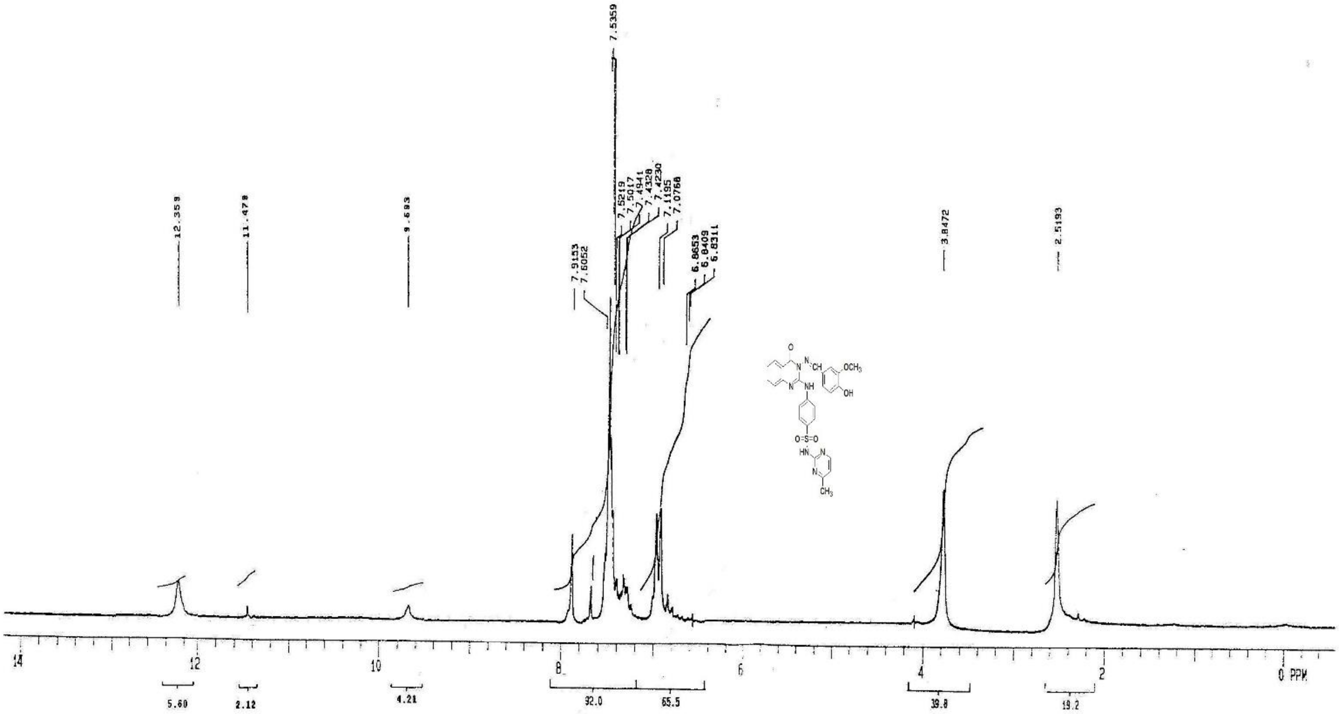

Figure S8: ^{1}H-NMR spectrum of comound **5**.

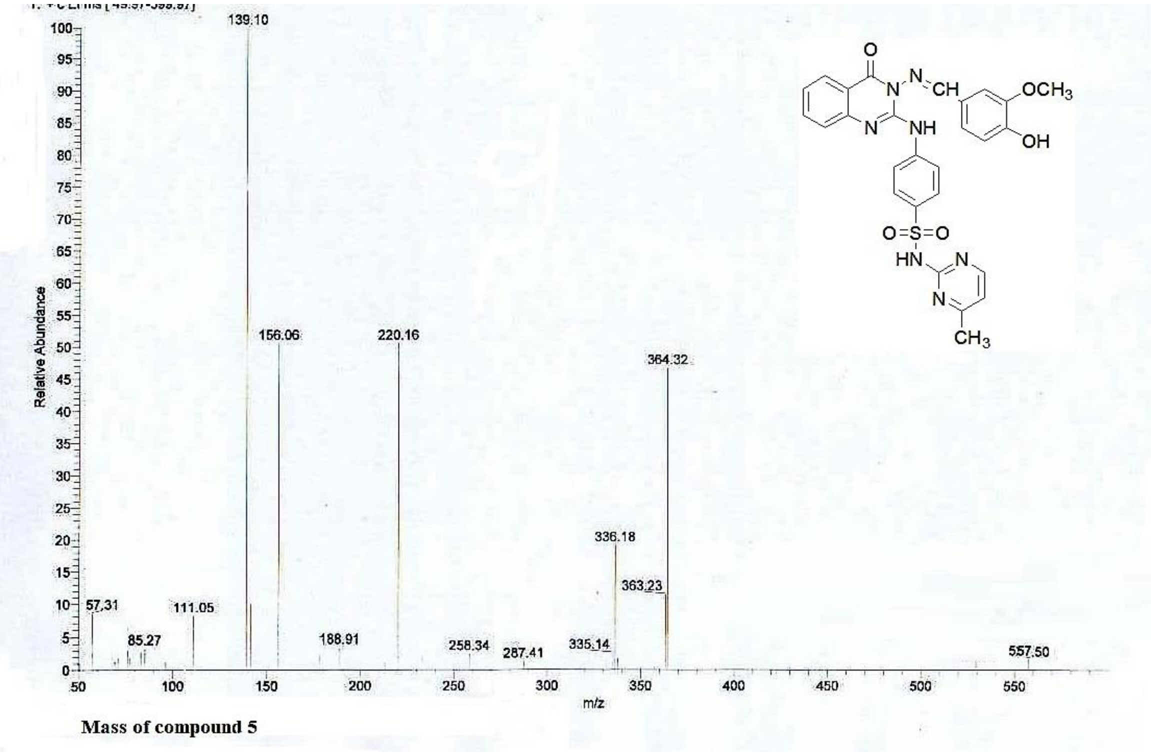

Figure S9: Mass spectrum of comound **5**.

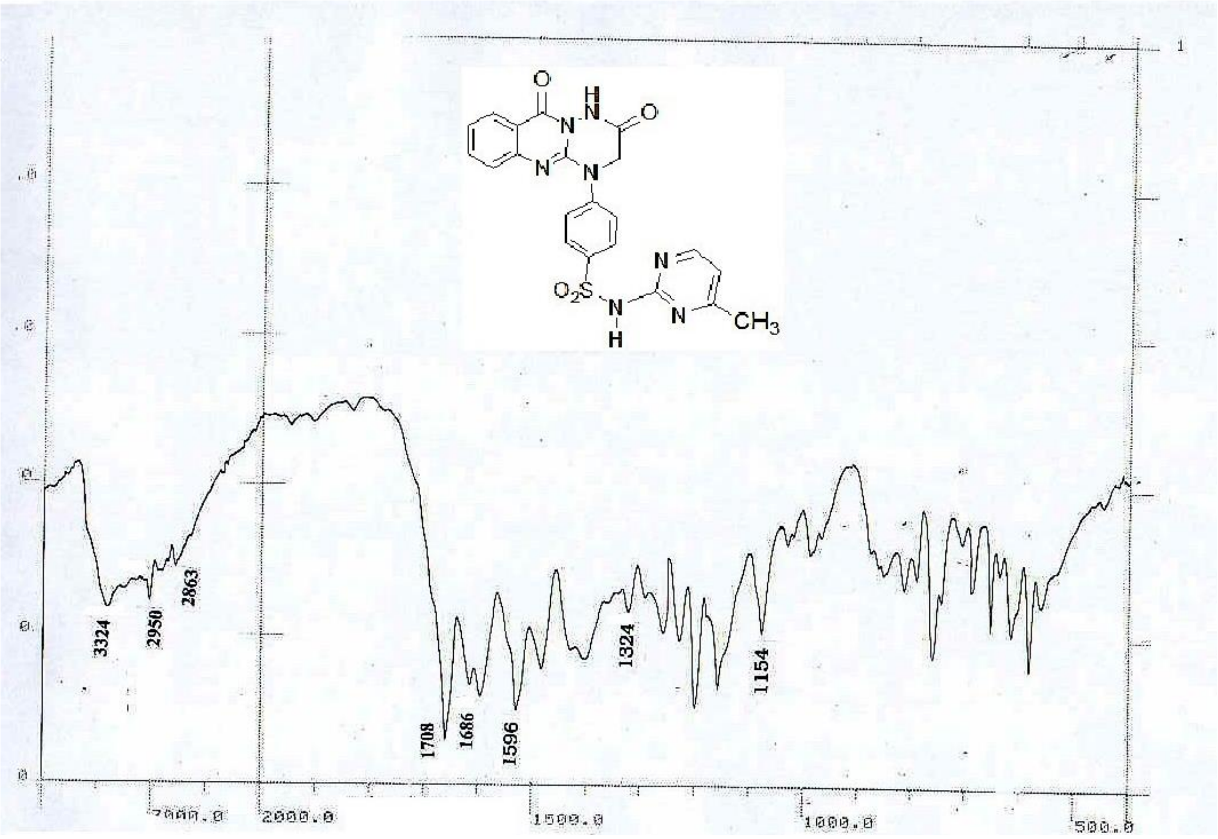

Figure S10: IR spectrum of comound **6**.

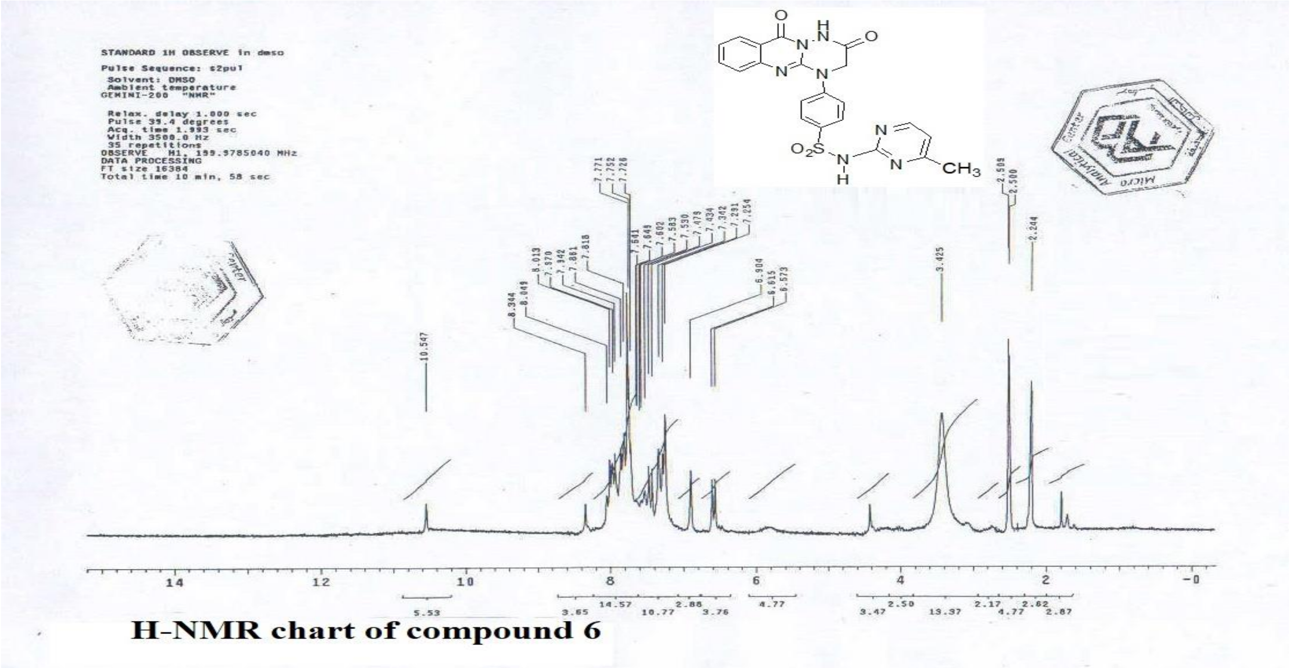

Figure S11: ^{1}H-NMR spectrum of comound **6**.

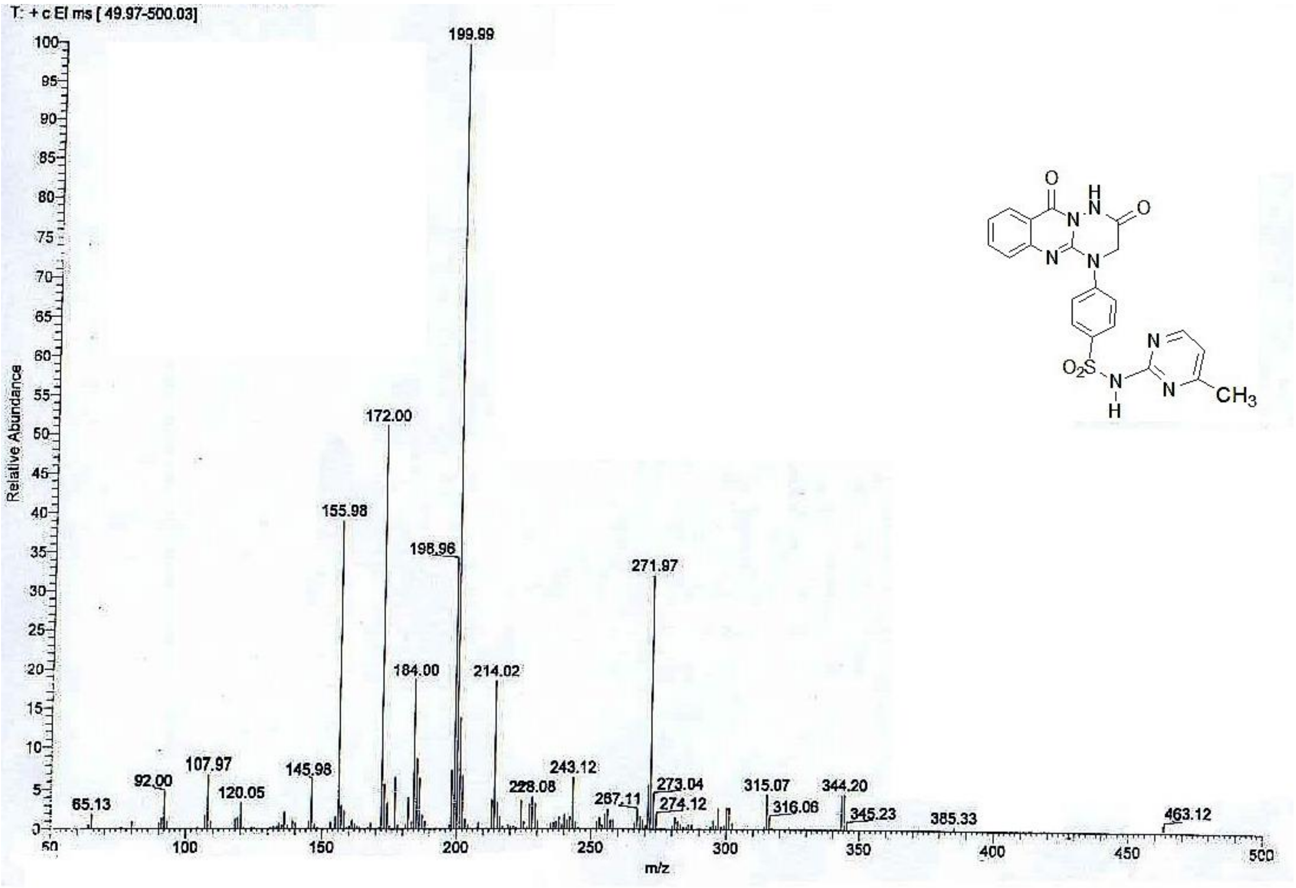

Figure S12: Mass spectrum of comound **6**.

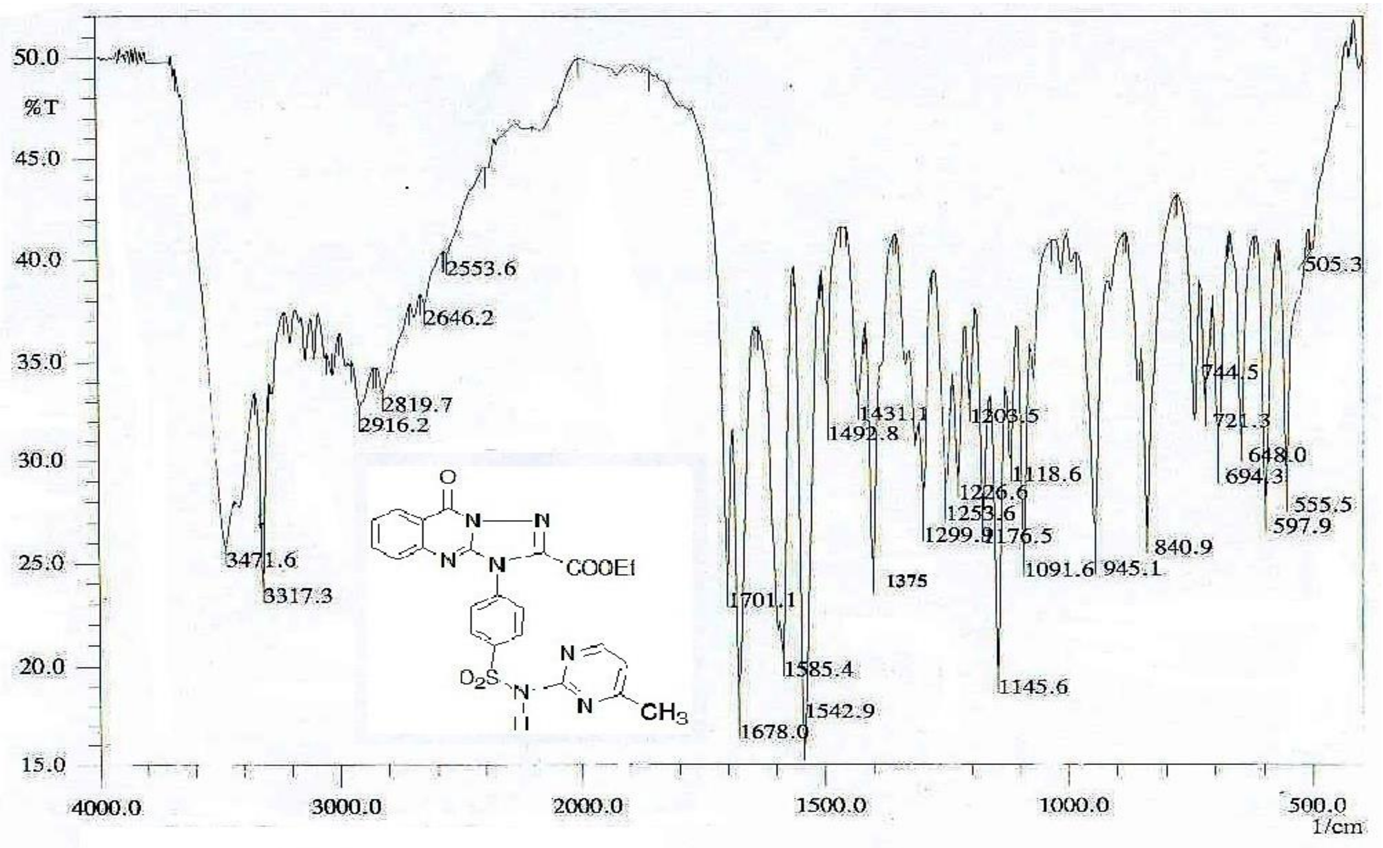

Figure S13: IR spectrum of comound **12**.

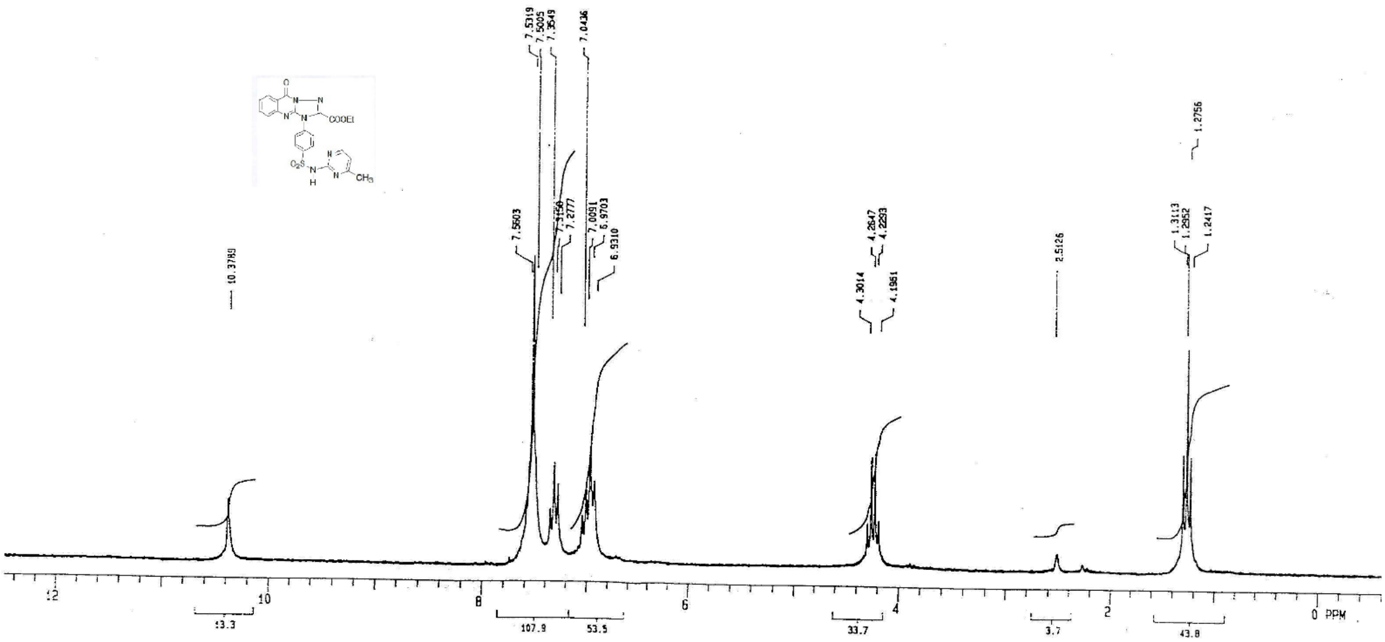

Figure S14: ^{1}H-NMR spectrum of comound **12**.

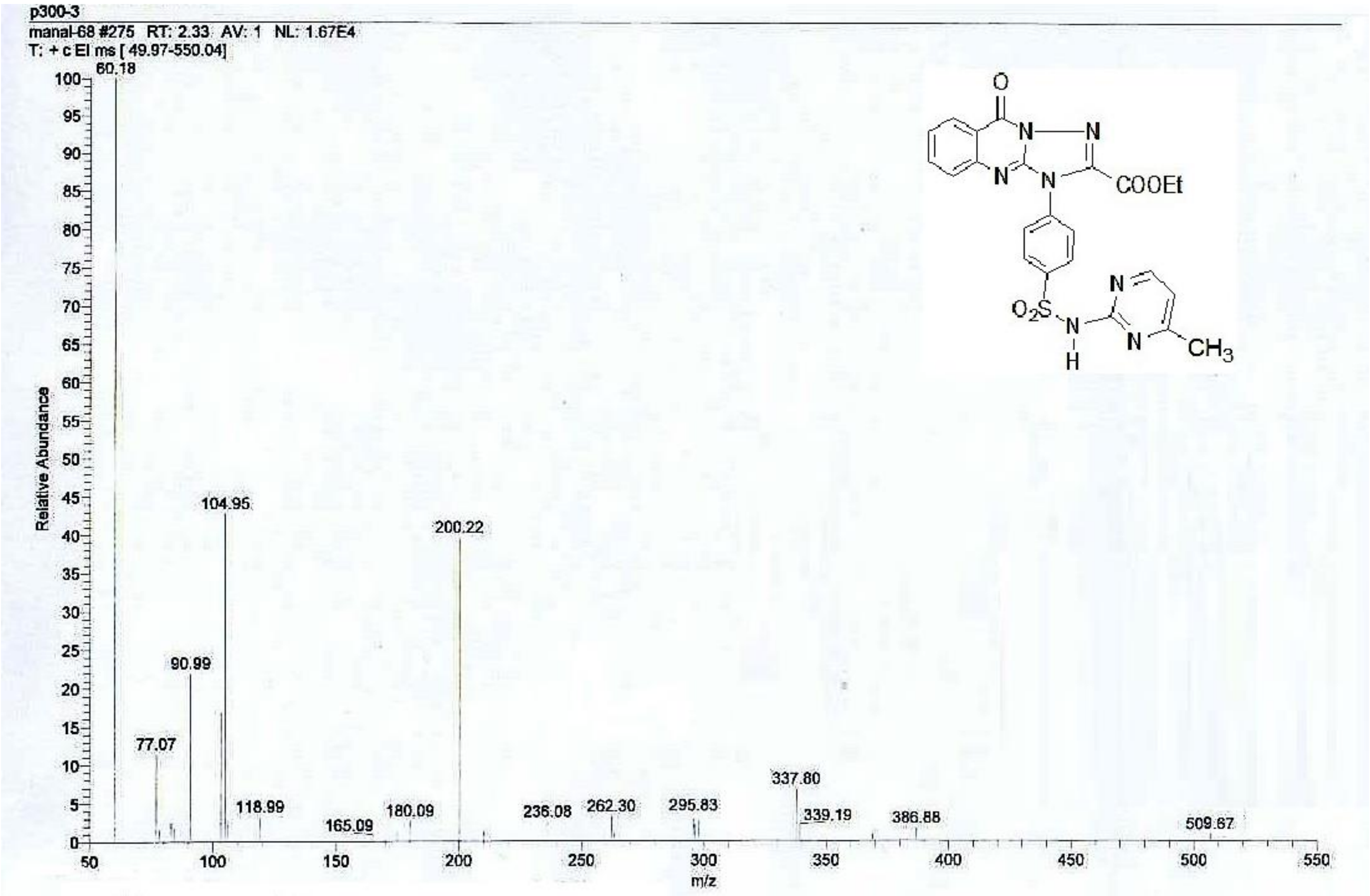

Figure S15: Mass spectrum of comound **12**.
