# Supplementary material for: Structure Activity Relationship and Molecular Docking of Some Quinazolines Bearing Sulfamerazine Moiety as New 3CLpro, cPLA2, sPLA2 Inhibitors
Source: Molecules. 2023 Aug 14;28(16):6052. doi: 10.3390/molecules28166052 (PMC10460087; doi:10.3390/molecules28166052)
Supplement: Supplementary file 1 [file molecules-28-06052-s001.zip › Tables S1 and S2.pdf]

Table S1: IR and ^{1}H-NMR spectral data of compounds **3–6** and **12**.

Compounds	IR	^{1}H-NMR	Mass spectra
**3**	IR (KBr, cm^{-1}) **3**: 3420, 3380, 3150(3NH), 1660(C=O), 1610(C=N), 1350,1160 (SO_2), 1240 (C=S).	^{1}H-NMR (DMSO-d_6) **3**: δ 2.50 (s) 3H, CH_3; δ 3.81, 3.83, 3.86 (t) 3H, OCH_3; δ 6.9-8.17(m) 8H, Ar H + 2H-pyrimidine; δ 10.3 and 11.6 (2s) 2H, 2NH; δ 13.14 (s) 1H, SO_2NH.	MS (m/z): 457(M^+, 1.70%), 415.28 (2.54%), 287.09(100%), 228.04 (15.76%), 194.02 (58.23%), 77.02(10.7%), 77 (94.24%).
**4**	IR(KBr, cm^{-1}) **4**: 3310, 3205, 3097 (NH, NH_2), 2993, 2889 (CH-aliph.) 1685(C=O), 1593(C=N), 1407,1157(SO_2).	^{1}H-NMR (DMSO-d_6) **4**: δ 2.4 (s) 3H, CH_3; δ 5.6 (s) 2H, NH_2; δ 6.0-8.00(m) 8H, Ar H + 2H-pyrimidine; δ 11.6 (s) 1H, NH; δ 13.11 (s) 1H, SO_2NH.	MS (m/z) : 423(M+, 3.45%), 380 (8.64%), 327(2.74%), 240 (100%), 223 (36.6%), 120(20.08%), 65(93.3%), 77 (94.24%).
**5**	IR (KBr, cm^{-1}) **5**: 3402 (NH), 3232(CH-arom.), 2985, 2842 (CH-aliph.), 1620(C=O), 1326, 1180(SO_2).	^{1}H-NMR (DMSO-d6) **5**: δ 2.5 (s) 3H, CH_3; δ 3.84 (s) 3H, CH_3; δ 6.8-7.91(m) 11H, Ar H + 2H-pyrimidine; δ 9.69 (s) 1H, NH; δ 11.47 (s) 1H, C=NH; δ 12.35 (s) 1H, SO_2NH.	MS (m/z): 557(M+, 3.5%), 364.32 (49.8%), 336.18(22.6%), 220.16 (51.70%),156.06 (54.6%), 139(100 %), 111(9.6%), 85.27 (4.5%), 57.31(9.8%).
**6**	IR (KBr, cm^{-1}) **6**: 3324 (NH)), 2950 (CH-arom.), 2863 (CH-aliph.), 1708, 1686 (2C=O), 1324, 1154(C=N), 1346, 1145(SO_2).	^{1}H-NMR (DMSO-d_6) **6:** revealed signals at 2.3[s, 3H, CH_3- pyrimidine], 4.4 [s, 2H, CH_2], 7.2 – 8.0 [m, 11H, Ar-H], 8.3 [m, 1H, NH], 10.54[s, 1H, SO_2NH].	MS (m/z) : 463(M+, 2.1%), 385.3 (1.9%), 344.2(4.8%), 315.07 (6.1%),271.97 (33.4%), 199.9(100 %), 172.00(52.6%), 155.98 (42.8%), 92.0(5.1%), 65.13 (3.1%).
**12**	IR (KBr, cm^{-1}) **12**: 3471, 3317 cm^{-1}(2NH), 2916(CH-arom.), 2931 (CH-aliph.), 1701, 1678 cm^{-1}(2C=O), 1542 cm^{-1} (C=N), 1375, 1145 cm^{-1}(SO_2).	^{1}H-NMR (DMSO-d_6) **12**: 1.24, 1.29, 1.31[t, 3H, CH_3], 2.51[s, 3H, CH_3-pyrimidine], 4.19, 4.11, 4,26, 4.30 [q, 2H, CH_2], 6.93– 7.56[m, 8H, Ar-H and 2H, pyrimidine], 10.37[s, 1H, SO_2NH].	MS (m/z) 12: 509.67(M+, 2.3%), 386.88(3.1%), 337.80(7.4%), 295.8 (6.5%),262.30 (4.2%), 200.22(46.7 %), 180.09(3.8%), 104.95 (48.3%), 90.99(22.5%), 77.07 (12.7%), 60.18 (100 %).

Table S2. Characteristic data for synthesized compounds.

**Compd. No.**	**M.P. [°C]**	**Yield (%)**	Mol. Formula (Mol. Wt.)	Elemental analyses Calcd./Found [%] C	H	N
**3**	**279-281**	**74**	$C_{20}H_{19}N_5O_4S_2$	52.51	4.16	15.32
			(457)	52.41	4.35	15.55
**4**	**205-207**	**77**	$C_{19}H_{17}N_7O_3S$	53.90	4.02	23.16
			(423)	53.80	4.20	23.27
**5**	**150-152**	**69**	$C_{27}H_{23}N_7O_5S$	56.04	4.12	17.59
			(557)	56.35	4.20	18.00
**6**	**201-203**	**68**	$C_{20}H_{15}N_7O_4S$	54.42	3.67	21.17
			(463)	55.00	3.50	21.40
**12**	**110-112**	**81**	$C_{22}H_{17}N_7O_5S$	54.40	3.50	20.30
			(505)	54.80	3.40	20.20
